# Mammary Paget’s disease in a young woman: A rare occurrence

**DOI:** 10.1016/j.radcr.2025.04.062

**Published:** 2025-05-15

**Authors:** Chaimae Abourak, Aya Laridi, Ouafaa Chahboune, Siham Oukassem, Asmae Guennouni, Soukaina Bahha, Lina Belkouchi, Nazik Allali, Latifa Chat, Siham El Haddad

**Affiliations:** Department of Radiology, Mother-Child, Faculty of Medicine and Pharmacy of Rabat, Children's Hospital, Ibn Sina University Hospital, Mohammed V University, Rabat, Morocco

**Keywords:** Mammary Paget's disease, MPD, Paget cells, MRI

## Abstract

Mammary Paget's disease (MPD) is a rare intraepithelial carcinoma involving the nipple-areolar complex, often linked to underlying ductal carcinoma. We report a case of a 47-year-old woman presenting with progressive swelling of the left nipple, without pain, discharge, or inflammatory signs. Imaging revealed ductal dilation and nipple thickening, classified as BI-RADS 4, with MRI confirming additional suspicious findings. Histopathological analysis of a biopsy confirmed MPD associated with underlying ductal carcinoma in situ. This case underscores the importance of multimodal imaging and histopathology in diagnosing and managing this uncommon breast malignancy.

## Introduction

Mammary Paget’s disease (MPD) is a rare intraepithelial malignancy of the nipple–areolar complex, often clinically mistaken for other benign or malignant breast conditions [1]. It is considered an uncommon manifestation of breast cancer and is histopathologically characterized by the presence of Paget cells [2]. Although more commonly observed in postmenopausal women, MPD can also affect younger patients, in whom diagnosis is often delayed due to lower clinical suspicion and its resemblance to benign dermatological conditions. In these cases, imaging plays a crucial role in early detection by identifying underlying malignancies and guiding further management.

### Case report

**Fig. 1:**
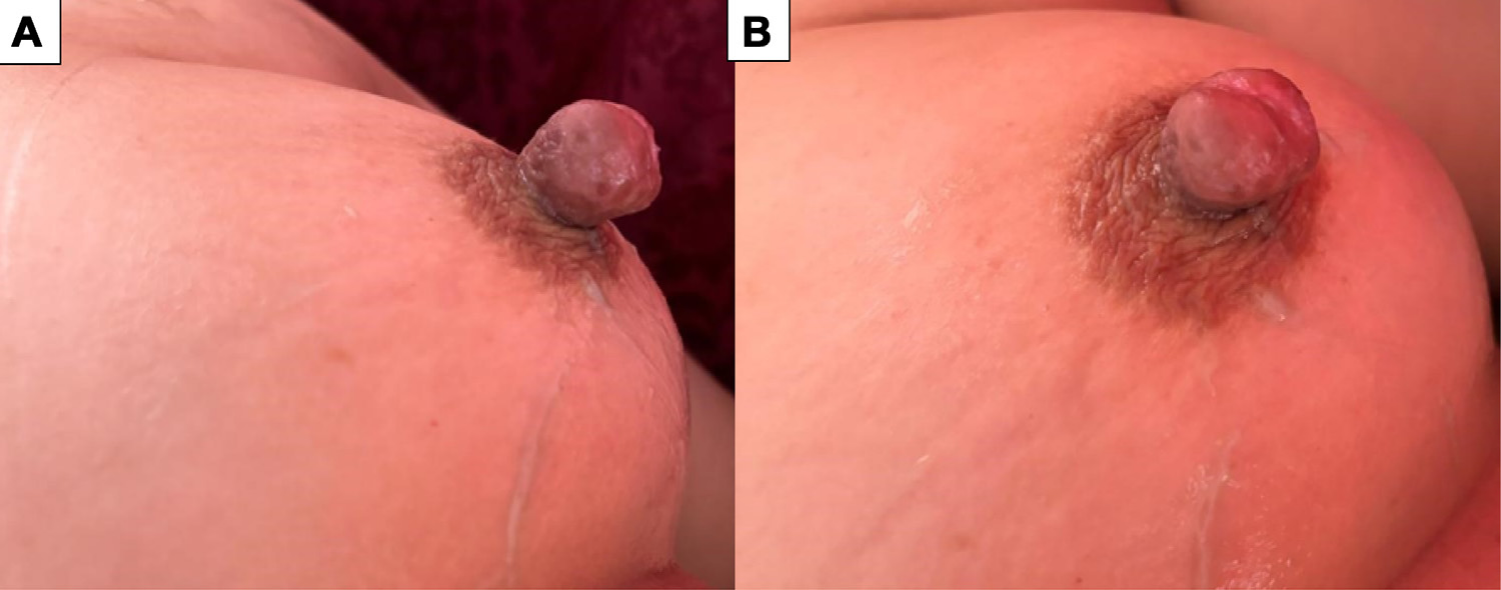
(A and B) Clinical images showing a swollen left nipple without nipple discharge or associated inflammatory signs.

We report the case of a 47-year-old woman, mother of 2, with no significant medical history, who presented with a progressively enlarging swelling of the left nipple, without any associated symptoms. Clinical examination revealed that she was hemodynamically, respiratory, and neurologically stable. Breast examination showed a nonpainful swelling of the left nipple (Fig. 1), without local inflammatory signs, nipple discharge, or other visible skin abnormalities. Palpation of both breasts revealed no palpable masses, and no axillary lymphadenopathy was detected.

**Fig. 2:**
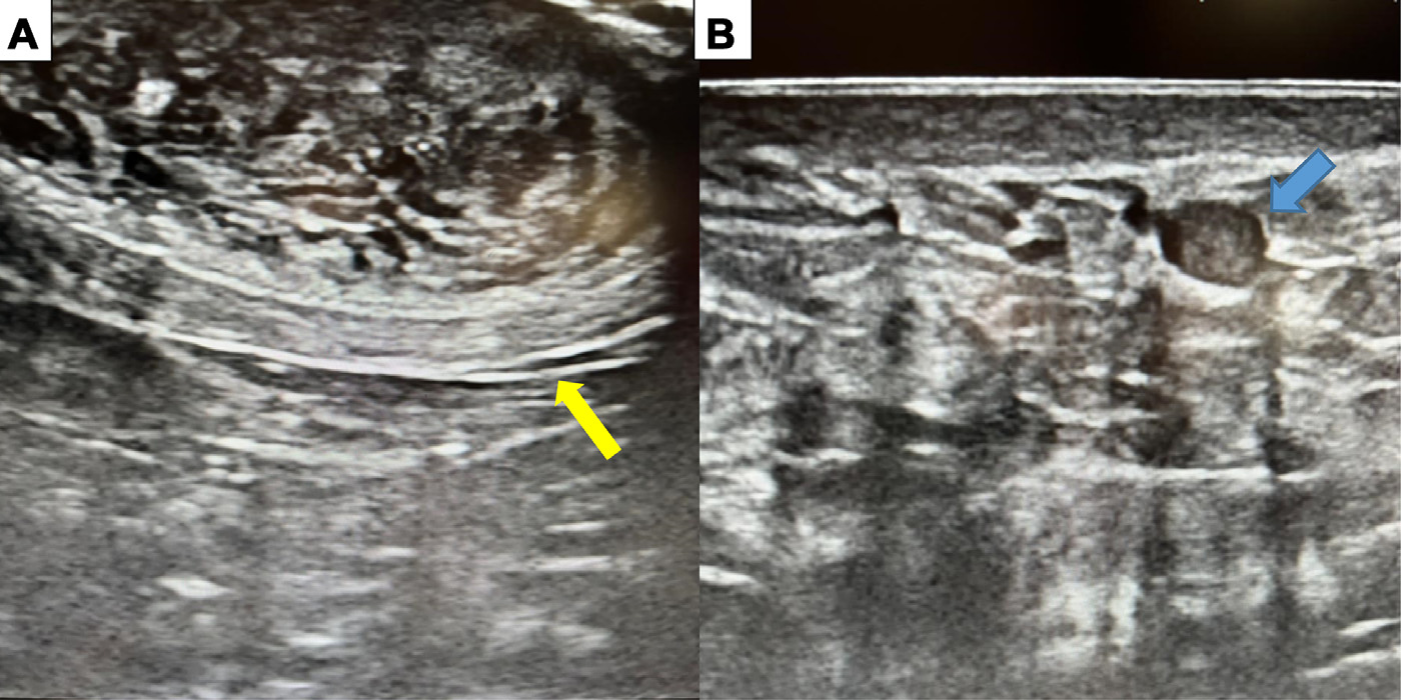
Images of a breast ultrasound of the left breast; heterogeneous and swollen appearance of the nipple (A) (yellow arrow), focal ductal dilation in the inferolateral quadrant showing a tissue-like projection (B) (blue arrow).

A breast imaging workup was initiated, including ultrasound examination. The ultrasound revealed a focal ductal dilatation in the left breast, containing an intraductal tissue-like projection, associated with nipple thickening (Fig. 2A). These findings were classified as BI-RADS 4 according to the ACR, indicating a suspicion of malignancy. Additionally, an isolated intramammary lymph node was identified in the right breast, classified as BI-RADS 2, consistent with a benign lesion (Fig. 2B).

**Fig. 3:**
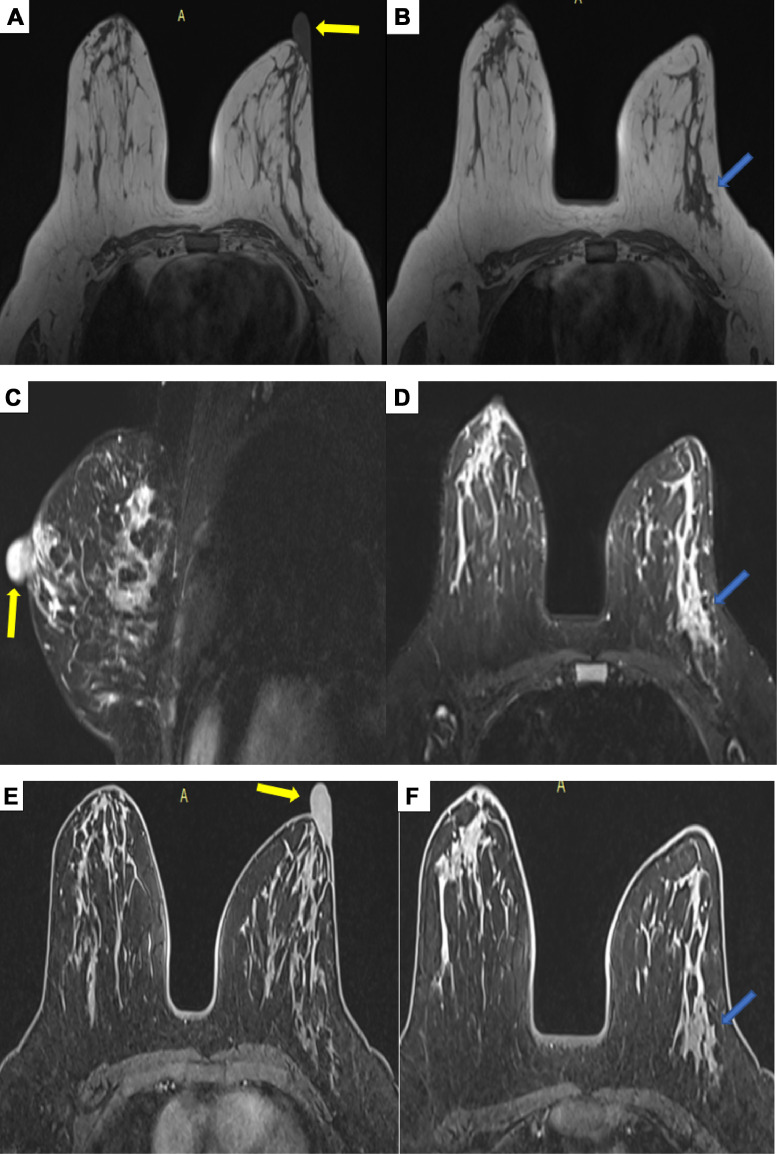
Breast MRI. Axial T1 sequences without contrast injection (A and B), T2 Dixon sequences with fat saturation, sagittal cut (C) and axial cut (D), T1 postcontrast sequences with fat saturation, axial cuts (E and F). Finely heterogeneous thickening of the left nipple, enhanced following contrast injection, measuring 24 × 16 mm (yellow arrow). Furthermore, a tubular lesion was observed in the inferolateral quadrant of the left breast. This lesion, aligned in a ductal orientation, appeared isointense on T1-weighted sequences and hyperintense on T2-weighted sequences, with enhancement following a type 3 curve (rapid uptake followed by washout), characteristic of suspicious lesions. The lesion measured 5.5 × 5.5 × 13 mm (blue arrow).

To further characterize the abnormalities, a breast MRI was performed. The imaging revealed a finely heterogeneous thickening of the left nipple, measuring 24 × 16 mm, with postcontrast enhancement. Additionally, a tubular lesion was identified in the inferolateral quadrant of the left breast. This lesion, aligned along a ductal axis, appeared isointense on T1-weighted sequences and hyperintense on T2-weighted sequences, with postcontrast enhancement showing a type 3 kinetic curve (rapid initial uptake followed by washout), which is characteristic of suspicious lesions. The lesion measured 5.5 × 5.5 × 13 mm (Fig. 3).

The combined clinical and radiological findings strongly suggested Paget’s disease of the breast, likely associated with underlying ductal involvement. An ultrasound-guided biopsy was performed to confirm the diagnosis. Histopathological examination confirmed the diagnosis of Paget’s disease of the breast. The presence of Paget cells, characterized by clear, mucin-rich cytoplasm and pleomorphic, hyperchromatic nuclei, further corroborated the diagnosis.

## Discussion

MPD, first described by Sir James Paget in 1874, is a rare condition presenting as an eczematous lesion on the nipple, often associated with an underlying malignancy¹. It is now recognized as a cutaneous intraepithelial carcinoma characterized by the presence of large adenocarcinoma cells, known as Paget’s cells, within the squamous epithelium of the nipple. These cells may also spread to the areola and surrounding skin [2].

MPD primarily affects postmenopausal women, typically in their 50s or 60s, and is exceptionally rare in younger women [3], making this one of the few documented cases of MPD in a younger patient. Diagnosis in this population is particularly challenging due to lower clinical suspicion and overlapping presentations with benign dermatological conditions.

MPD shares common breast cancer risk factors, including advanced age, alcohol use, obesity, BRCA mutations, chest radiation, hormone therapy, contraceptive use, and family history. A study by Zheng et al. found that MPD and other breast cancers exhibit similar demographic and risk profiles [4]. However, nulliparity is more frequent among MPD patients [5].

MPD accounts for approximately 1% to 3% of all breast cancer cases. Over 90% of MPD cases are associated with underlying ductal carcinoma in situ (DCIS) or invasive ductal carcinoma (IDC), which are often centrally located or multifocal [6].

The pathogenesis of MPD remains debated, with 2 main theories proposed. The widely accepted epidermotropic theory suggests that Paget cells originate from underlying breast adenocarcinoma and migrate to the nipple epidermis. This theory is strongly supported by immunohistochemical studies demonstrating frequent HER2 overexpression in both Paget cells and the associated carcinoma. HER2-positive status is observed in 80%-100% of MPD cases, further reinforcing the idea that Paget cells share a common origin with the underlying malignancy.

The less widely accepted intraepidermal transformation theory posits that Paget cells arise independently through malignant transformation of keratinocytes or apocrine cells. Despite extensive research, direct evidence for either theory is lacking, though the epidermotropic theory is more widely endorsed [6].

MPD can be classified into 3 distinct categories based on the presence or severity of associated disease: MPD of the nipple without accompanying DCIS, MPD of the nipple with DCIS confined to the underlying lactiferous ducts within 2 cm of the nipple, and MPD of the nipple with DCIS in the underlying lactiferous ducts, accompanied by either additional DCIS or invasive breast cancer located elsewhere in the breast, extending 2 cm or more from the nipple-areolar complex [7].

MPD is defined histopathologically by the presence of Paget cells, which are malignant epithelial cells characterized by clear, abundant cytoplasm rich in mucin and pleomorphic, hyperchromatic nuclei. These cells typically form nest-like or gland-like structures within the basal layer of the epidermis and may completely replace epidermal cells. In advanced stages, invasion of adnexal structures can occur, along with dermal changes such as telangiectasia, chronic inflammation, and ulceration. Immunohistochemistry plays a crucial role in diagnosis, showing overexpression of CK7 and the absence of CK10, CK14, and CK20. Paget cells share immunohistochemical features with the associated breast cancer cells, often lacking estrogen and progesterone receptor expression. They also exhibit markers such as p53, p21, Ki-67, cyclin D1, androgen receptors, and Her-2 oncoprotein [8].

Regarding clinical presentation, MPD typically manifests with unilateral dermatological changes in the nipple–areolar complex, including pruritus, eczema, erythema, and, in advanced cases, skin erosion, ulceration, and crusting. MPD is almost always unilateral, in contrast to noncancerous eczema, which typically affects both sides. Physical symptoms may include pain or a burning sensation, with lesions often spreading to adjacent areas. Misdiagnosis as dermatitis or eczema is common. Clinical studies show that most patients exhibit nipple masses, redness, and itching, with axillary lymph node involvement frequently observed in advanced stages [6,7,9].

MPD can closely resemble conditions such as dermatitis, eczema, erosive adenomatosis, Bowen’s disease, and melanoma, particularly in younger patients, leading to delayed diagnosis [8]. Benign conditions like ductal ectasia or nipple fissures may also be mistaken for MPD. Misdiagnosis as a noncancerous skin disorder increases the risk of disease progression. Persistent or worsening skin changes should prompt further evaluation. Imaging and biopsy are essential for accurate diagnosis and timely management.

Mammography is essential for diagnosing and managing Paget’s disease, although it may overlook abnormalities in up to 50% of cases. Typical findings include skin thickening near the nipple, variations in density, nipple retraction, masses, or microcalcifications [7]. Studies, such as that by Pelorca et al., report mammographic abnormalities in 87.7% of cases, with microcalcifications being the most common finding [10]. However, mammography can sometimes fail to detect malignancies, with reports of hidden cancers ranging from 15% to 65% [6]. Its sensitivity is particularly limited in younger women with dense breast tissue, which reduces its effectiveness as a standalone diagnostic tool. Despite these limitations, mammography remains valuable for monitoring patients who have undergone conservative breast surgery.

Ultrasound serves as a complementary tool to mammography, particularly when no abnormalities are visible on the latter. It can identify features such as parenchymal heterogeneity, hypoechoic areas, masses, dilated ducts, cutaneous thickening, and axillary lymph node involvement [11]. Although ultrasound does not detect microcalcifications, it enhances the visualization of masses, guides biopsies, and aids in lesion characterization. While it does not increase sensitivity when combined with mammography, it provides valuable additional characterization of the findings.

MRI is particularly effective in identifying breast tumors, especially when other imaging techniques yield inconclusive results. It can accurately detect thickening of the nipple–areolar complex, nipple enlargement, as well as both in situ and invasive carcinomas. Contrast-enhanced MRI is particularly useful in determining the extent of disease, especially for patients considering breast-conserving surgery. However, its high sensitivity is counterbalanced by lower specificity, which can lead to overdiagnosis or unnecessary interventions. MRI is superior for detecting multicentric and hidden lesions but may contribute to overdiagnosis. Despite these limitations, MRI often reveals hidden or multicentric lesions that are not detected through clinical examination or other imaging modalities [6,7].

Poor outcomes are associated with palpable breast tumors, lymph node involvement, invasive histology, and younger patients. Survival rates decline significantly with lymph node metastases, and male patients have a worse prognosis [8].

Mastectomy remains the standard treatment for MPD; however, conservative options, such as partial or total nipple excision, central segmentectomy, or radiotherapy, are emerging for cases confined to the central breast. These alternative treatments require close follow-up, including regular imaging. Additional therapies depend on tumor staging and lymph node metastases.

## Conclusion

Mammary Paget’s disease is an uncommon and often misdiagnosed form of breast cancer. A multidisciplinary approach, involving radiologists, pathologists, and oncologists, is essential for accurate diagnosis and optimal management. Imaging plays a crucial role in early detection, with mammography, ultrasound, and MRI assisting in the identification of underlying malignancies. Additionally, histopathological confirmation through biopsy is vital for establishing the diagnosis and guiding appropriate treatment, ultimately improving patient outcomes.

## Ethics approval

Our institution does not require ethical approval for reporting individual cases or case series.

## Patient consent

Written informed consent was obtained from the patient(s) for their anonymized information to be published in this article.
